# An Innovative Treatment Based on Sodium Citrate for Improving the Mechanical Performances of Flax Fiber Reinforced Composites

**DOI:** 10.3390/polym13040559

**Published:** 2021-02-13

**Authors:** Vincenzo Fiore, Dionisio Badagliacco, Carmelo Sanfilippo, Riccardo Miranda, Antonino Valenza

**Affiliations:** Department of Engineering, University of Palermo, Viale delle Scienze, Edificio 6, 90128 Palermo, Italy; dionisio.badagliacco@unipa.it (D.B.); carmelo.sanfilippo01@unipa.it (C.S.); riccardo.miranda@unipa.it (R.M.); antonino.valenza@unipa.it (A.V.)

**Keywords:** natural fibers, flax, chemical treatment, sodium citrate, fiber–matrix adhesion, mechanical properties

## Abstract

The goal of this paper is to evaluate the effectiveness of a cost-effective and eco-friendly treatment based on the use of sodium citrate (Na_3_C_6_H_5_O_7_) on the mechanical properties of flax fiber reinforced composites. To this scope, flax fibers were soaked in mildly alkaline solutions of the sodium salt at different weight concentration (i.e., 5%, 10% and 20%) for 120 h at 25 °C. The modifications on fibers surface induced by the proposed treatment were evaluated through Fourier transform infrared analysis (FTIR), whereas scanning electron microscope (SEM) and helium pycnometer were used to obtain useful information about composites morphology. The effect of the concentration of the treating solution on the mechanical response of composites was determined through quasi-static tensile and flexural tests, Charpy impact tests and dynamical mechanical thermal (DMTA) tests. The results revealed that composites reinforced with flax fibers treated in 10% solution exhibit the best mechanical performances as well as the lowest void contents. SEM analysis supported these findings showing that, by treating fibers in solutions with concentration up to 10%, composites having better morphology can be manufactured, in comparison to untreated ones. Conversely, higher Na_3_C_6_H_5_O_7_ concentrations negatively affect both the morphology and the mechanical properties of composites.

## 1. Introduction

Natural fiber reinforced composites (NFRCs) have received great attention in these last decades both from academia and from various industries. Due to their fully or partially biodegradability, light weight and cost-effectiveness, these polymeric composites have found application in several engineering fields such as automotive, marine and sport equipment’s.

However, a critical factor associated with these materials is the generally observed lower mechanical performance in comparison to their synthetic counterparts (i.e., glass or carbon fiber reinforced composites), mainly due to the weak fiber–matrix adhesion. This drawback is related to the hydrophilic (i.e., polar) and hydrophobic (i.e., non-polar) nature of natural fibers and epoxy matrices, respectively [1]. In particular, natural fibers are mainly composed by cellulose, hemicellulose and lignin. Lignin is an irregular polyphenolic polymer and presents a compact and hydrophobic structure. On the other hand, polysaccharide components, such as cellulose and hemicellulose have a large amount of strongly polarized hydroxyl groups, thus giving hydrophilic nature to the fiber. When these fibers are used as reinforcement of hydrophobic polymeric matrices such as epoxies, the resulting composites are inherently characterized by weak fiber–matrix adhesion that reduces the ability to transfer stress from matrix to fibers and, therefore, decreases their mechanical properties.

To overcome this issue, several physical [2,3,4] and chemical treatments [5,6,7] have been widely performed on natural fibers in order to modify their surface, thus enhancing the adhesion with polymeric matrices. Among chemical methods, the most common one is the mercerization that consists in the soaking of the fibers in a highly alkaline bath such as sodium hydroxide solution. As widely known, the surface of natural fibers during mercerization can be altered following the scheme:

Fiber-OH + NaOH → Fiber-O-Na^+^ + H_2_O (1)

Overall, the main effects of the mercerization consist in: (i) fiber fibrillation with consequent diameter reduction and aspect ratio increment; (ii) development of a rougher surface; (iii) increase of possible reactive sites and (iv) removal of cementing substances such as lignin and hemicellulose.

All these factors can contribute to improve the fiber–matrix compatibility, leading to noticeable increments of the performances of the resulting composites, as widely evidenced by several authors [8,9,10].

Nevertheless, it is worth noting that this method also presents some drawbacks. In particular, it can be considered an expensive and harmful to environment approach due to the use of hazardous chemical such as sodium hydroxide [11,12]. Moreover, such a highly alkaline treatment can reduce mechanical properties of the fibers, thus negatively impacting their suitability as reinforcement of composite structures [8,13,14,15]. Due to these reasons, new approaches based on eco-friendly and inexpensive compounds such as sodium bicarbonate and sodium carbonate have been studied in recent years [11,12,16,17,18,19,20,21,22]. These methods are based on the principle that by soaking natural fibers in a mildly alkaline solution having sodium cations, the interaction is similar to what happens during mercerization (Equation (1)).

It is worth noting that, due to the mildly alkaline environment achieved by using a weak basis, longer soaking times and/or higher concentration of the treating solution are required in comparison to the traditional mercerization [11,12,21].

In such a context, the present paper aims to evaluate the feasibility of using a mildly alkaline compound having sodium cations (i.e., sodium citrate) for treatment of natural fibers, in order to improve the compatibility between flax fibers and epoxy resin.

Sodium citrate is an inexpensive, widely available and non-toxic salt of citric acid, which can be used in medicine as source of sodium ions in locking solutions alternative to unfractionated heparin for dialysis catheters [23]. Being a sodium salt of a low molecular weight organic acid, it can be also used as food additive to extend the shelf life, as well as to improve the sensory attributes of meat [24] and fish [25,26], due to its antibacterial activities against various food-borne pathogens [27]. Furthermore, sodium citrate can be used in addition with appropriate organic inhibitors as practical scaling-corrosion inhibitor platform for protecting metal structures in seawater applications [28]. To the best of our knowledge, this inexpensive and safe sodium salt has not yet been used for improving the adhesion between natural fibers and polymeric matrices.

Starting from this premise, this study evaluates the effect of a cost-effective and eco-friendly treatment based on sodium citrate solution without adding any other chemical compounds on the properties of flax fiber reinforced composites. To this aim, flax fibers were treated with mildly alkaline solutions of this sodium salt at different weight concentration (i.e., 5%, 10% and 20%).

The chemical modifications on flax fibers were evaluated by means of Fourier transform infrared spectroscopy whereas scanning electron microscope (SEM) and helium pycnometer were used to investigate the morphology of the resulting composites. Furthermore, the effect of the concentration of the treating solution on the mechanical properties of composites was determined through quasi-static tensile and flexural tests, Charpy impact tests and dynamical mechanical thermal tests (DMTA). In order to identify significant differences among treatment conditions, a variance analysis (i.e., ANOVA) of the quasi-static mechanical results (i.e., tensile and flexural) was performed.

## 2. Experimental Section

### 2.1. Materials and Composites Manufacturing

The effectiveness of the proposed treatment was evaluated by varying the concentration of the treating solution. In particular, three solutions were prepared by adding 5%, 10% and 20% by weight of sodium citrate in demineralized water. Flax fibers (i.e., whole woven fabrics) were soaked in these solutions for 120 h at 25 °C. Afterwards, they were dried at room temperature for 24 h, and then oven dried (Enrico Bruno s.n.c., Torino, Italy) at 103 °C for further 24 h. Untreated fibers were also oven dried at the same conditions in order to remove their moisture content.

In total, four square panels (nominal length of 300 mm), reinforced with untreated and treated fibers (i.e., named R, C5, C10 and C20), were manufactured through vacuum assisted resin infusion process by using a two-stage vacuum pump model VE 235 D by Eurovacuum (Reeuwijk, The Netherlands). For each panel, six flax twill weave woven fabrics (Lineo, Valliquerville, France) with nominal areal weight 320 g/m^2^ were used as reinforcement and a DGEBA epoxy resin (SX8 EVO by Mates Italiana s.r.l., Segrate, Italy) mixed with its own amine-based hardener (100:30 by weight) was used as matrix. All the manufactured composites were cured at room temperature for 24 h and post-cured at 50 °C for 15 h. Specimens for each mechanical characterization were cut from the manufactured panels to their nominal dimensions as function of the specific test, by using a diamond blade saw.

**Figure 1:**
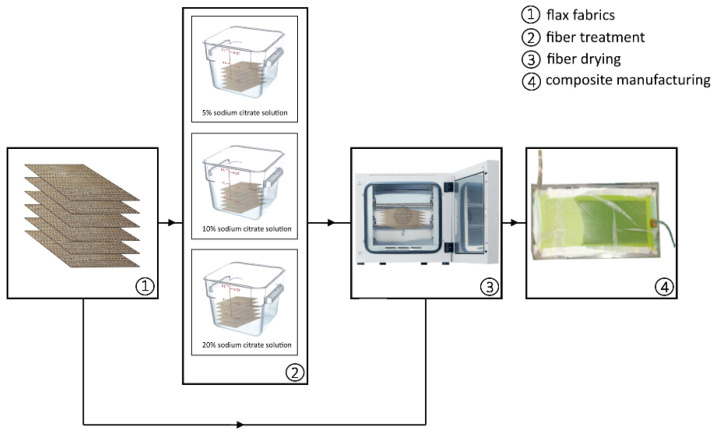
Flow diagram of the experimental procedure.

For the sake of clearness, the flow diagram of the experimental procedure is shown in Figure 1.

### 2.2. Mechanical Characterization

Quasi-static tensile tests were performed according to ASTM D3039 standard by means of an electromechanical Universal Testing Machine (U.T.M) model ETM-C by WANCE (Shenzhen, China), equipped with a load cell of 50 kN. A total of five specimens for each condition (25 mm × 250 mm) were tested in displacement control mode by setting the crosshead speed equal to 2 mm/min. The displacement was evaluated with the aid of an extensometer model YYU-10/50 by WANCE (Shenzhen, China), having gauge length 50 mm.

Quasi-static three-point bending tests were performed according to ASTM D790 standard using a U.T.M. model Z005 by Zwick-Roell (Ulm, Germany), equipped with a 5 kN load cell. In total, five specimens (15 mm × 90 mm) for each treatment condition were tested in displacement control mode at crosshead speed equal to 2 mm/min. Since the composites’ thickness varies in the range between 4.1 mm and 4.7 mm as function of the treatment condition, flexural tests were performed by keeping the span to thickness ratio equal to 16.

The quasi-static mechanical results were analyzed through a one-way Analysis of Variance (ANOVA) using Minitab^®^ software.

Dynamic mechanical thermal analysis (DMTA) was performed in tensile mode according to ASTM D4065 standard. A dynamic mechanical analyzer model DMA + 150 (Metravib, Limonest, France) was used to test three prismatic specimens (4 mm × 46 mm) for each condition from room temperature to 150 °C, at heating rate of 5 °C/min. The dynamic displacement and frequency were set equal to 1 × 10^−5^ m and 1 Hz, respectively.

Charpy impact tests were carried out in accordance with EN ISO 179 standard, with the aid of a pendulum model 9050 by CEAST (Pianezza, Italy). One impact energy level (5 J) was used throughout this study by impacting five prismatic specimens (80 mm × 10 mm) for each condition, at speed of 3.8 m/s.

### 2.3. Morphological Characterization

The void volume fraction (*ν_V_*) of each composite was evaluated by comparing its experimental and theoretical densities:

(2) $$\nu_{V}=\frac{\rho_{t}-\rho_{e}}{\rho_{e}}$$

The composites’ experimental density (*ρ_e_*) was evaluated through a helium pycnometer model Pycnomatic ATC by Thermo Electron Corporation (Waltham, MA, USA) and an analytical balance model AX 224 by Sartorius (Gottinga, Germany). For each sample, 10 measures were carried out and average values were recorded. All the measured standard deviations measured were lower than 0.01 g/cm^3^.

The theoretical density *ρ_t_* was calculated with the following equation:

(3) $$\rho_{t}=\frac{1}{\left(\frac{W_{f}}{\rho_{f}}\right)+\left(\frac{W_{m}}{\rho_{m}}\right)}$$

where *ρ_m_* is the experimental density of the epoxy matrix, equal to 1.1661 g/cm^3^. The experimental density values of flax fibers (*ρ_f_*) were measured for each treatment condition. *W_m_* and *W_f_* represent the weight content of epoxy resin and flax fibers, respectively.

The fractured surfaces of tensile specimens were observed through a SEM model Quanta 200F ESEM by FEI (Hillsboro, OR, USA). Each sample was sputter-coated with a thin layer of gold to avoid electrostatic charging under the electron beam and rubbed upon a 25 mm diameter aluminum disc.

Fourier transform infrared spectrometry (FTIR) was carried out on untreated and treated fibers in order to evaluate the effect of the proposed treatment on the chemical structure of their components. IR spectra of the fibers were recorded at the resolutions of 1 cm^−1^ using a Perkin Elmer (Waltham, MA, USA) spectrometer model Spectrum II in the frequency range 4000–500 cm^−1^, operating in attenuated total reflectance (ATR) mode.

## 3. Results and Discussion

### 3.1. Mechanical Characterization

**Figure 2:**
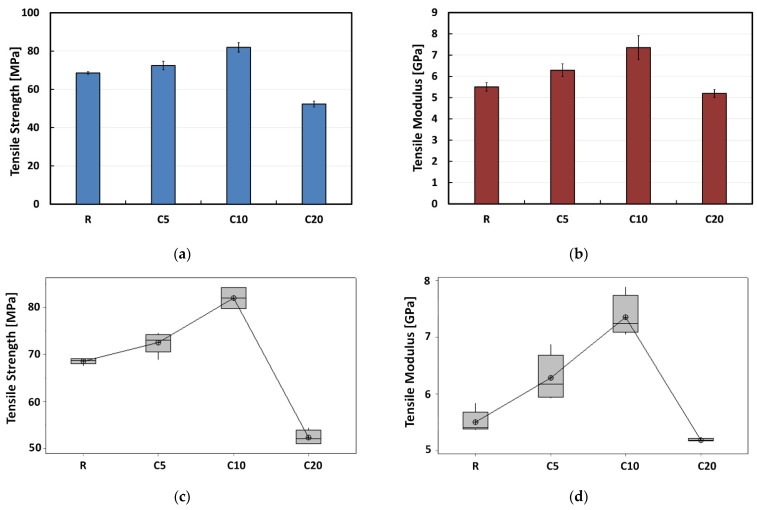
Tensile properties (**a**,**b**) bar chart and (**c**,**d**) box plot for each treatment condition.

The tensile properties of composites are shown in Figure 2. From Figure 2a,b it is possible to notice that the proposed treatment has beneficial effect on both tensile strength and modulus, for concentrations of the treating solution up to 10%. Indeed, the composites reinforced with treated flax fibers experience increments in the tensile strength equal to +5.7% and +19.5% (for treatments with 5 and 10 wt.% of sodium citrate, respectively) in comparison to the untreated one. Similarly, the tensile modulus increases by increasing the concentration of the treating solution up to 10%: i.e., +14.2% and +33.6% for the 5% and 10% treated composites (i.e., C5 and C10), respectively. Furthermore, the best tensile performances among all the investigated composites are evidenced by C10 composites that show average tensile strength and modulus values equal to 81.9 MPa and 7.35 GPa, respectively. Conversely, a worsening of the tensile properties can be noticed for composites reinforced with flax fibers treated with 20% sodium citrate solution. In particular, the average tensile strength and modulus of C20 composites are equal to 52.3 MPa and 5.19 GPa, respectively: i.e., −23.7% and −5.6% lower than untreated ones (i.e., R composites). As shown in Figure 2c,d, the box plots obtained from ANOVA evidence that the experimental data are statistically different, since the relative interquartile ranges (IQR) are totally detached.

**Figure 3:**
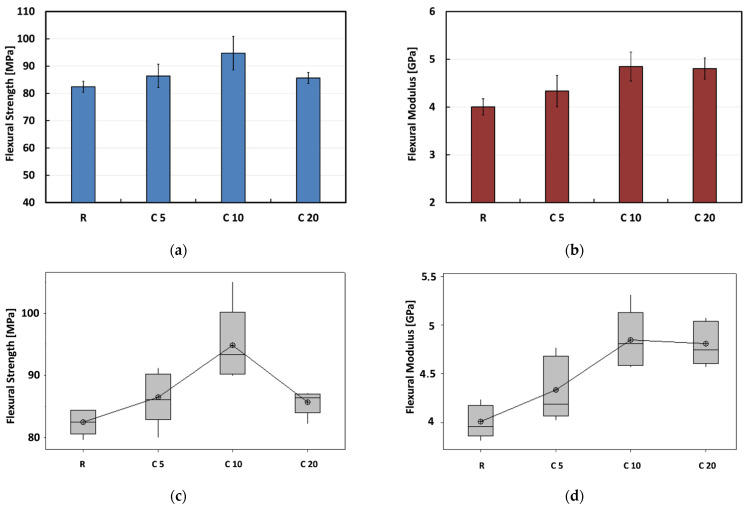
Flexural properties (**a**,**b**) bar chart and (**c**,**d**) box plot for each treatment condition.

Quite similar results are obtained from three-point bending tests, as shown in Figure 3. However, even in this case C10 composites show the best flexural performances. In particular, these composites experience increments in the flexural strength and modulus in comparison to untreated composites equal to +14.9% and +21.1%, respectively. By increasing the weight concentration of the treating solution to 20%, the tensile performances of composites tend to stabilize (i.e., modulus) or to decrease (i.e., strength). Nevertheless, C20 composites also show better flexural properties than untreated composites (i.e., +3.9% and +20% in the flexural strength and modulus, respectively).

**Figure 4:**
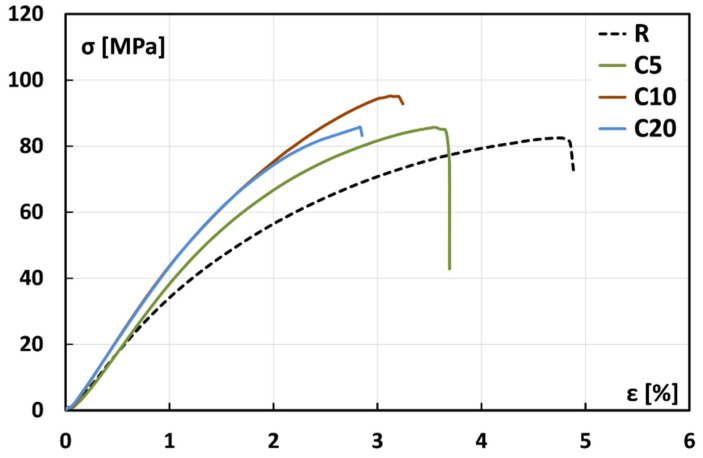
Typical stress-strain flexural curves of flax fiber reinforced composites.

The Analysis of Variance (ANOVA) has been performed to assess the statistical differences between the average values of tensile and flexural properties. The statistical results reported in Table 1 confirm the effectiveness of the chemical treatment on the improvement of the mechanical properties of the composites up to 10% of concentration. In particular, it is shown since the *p*-values are significantly lower than 0.05 for all the mechanical properties investigated. Further considerations can be acquired by observing the typical stress-strain flexural curves of composites, shown in Figure 4. It is possible to note that, regardless of the treatment condition, all the curves showed three different stages: (i) an initial stage up to about 1% strain in which samples evidence a linear elastic behavior, that allows the modulus measurement; (ii) a subsequent branch of non-linear trend corresponding to the material softening; (iii) a final stage showing a slighter decreasing trend in the curve slope until the sudden and catastrophic failure of the sample, which indicates the occurrence of a brittle failure. No noticeable plastic deformation can be evidenced after the stress drop: i.e., the crack propagates fast without any increase in the applied load when the samples reached the peak stress.

The highest values of the slope in the initial stage of stress-strain curve were observed for C20 and C10 composites, followed by C5 and R composites, thus evidencing the beneficial effect of the proposed treatment on the composites stiffness. Moreover, untreated composites showed the lowest flexural strength in comparison to the composites reinforced with treated fibers. In particular, C10 composites behave better than treated composites in terms of flexural strength, although C5 and C20 composites show higher and lower deformation at break in comparison to C10 composites, respectively. In particular, the final stage of the stress-strain curve of C20 composites is characterized by a noticeable slope decrement, due to the micro defects and micro-cracks that start to coalesce at increasing load levels, thus leading to a premature failure of the sample.

All these results evidence that the compatibility between epoxy matrix and flax fibers is noticeably improved after the sodium citrate treatment, for weight concentrations of the treating solution up to 10%. In particular, the increase in both tensile and flexural stiffness shown by C5 and C10 composites in comparison to the untreated one (i.e., R composites) can be explained taking into account that a stronger fiber–matrix adhesion results in more restrictive constraints on the relative displacements between fiber and matrix, particularly in the linear elastic phase [21]. This beneficial effect of the proposed treatment on the fiber–matrix adhesion also leads to an evident strengthening of the C5 and C10 composites, as shown by their tensile and flexural strength values, noticeably higher than that of untreated samples. A wide literature can be found concerning the increase in the tensile and flexural quasi-static strength of NFRCs materials due to the improved fiber–matrix compatibility after chemical treatment [9,29,30] of natural fibers.

Furthermore, the quasi-static mechanical results point out that the effectiveness of the proposed treatment is more evident on the tensile properties of the resulting composites in comparison to their flexural properties (see Figure 1 and Figure 2). As stated by other authors [9], this can be explained considering that the flexural failure mode shows less fiber pull-out phenomena, due to the applied stress direction, perpendicular to the composite sample in the three-point bending tests.

The improved mechanical response of the resulting composites can be also explained taking into account other effects due to the treatment of natural fibers. In particular, in addition to the strongest fiber–matrix adhesion, the mechanical properties enhancement could be also addressed to the lower void content inside the laminates, as a consequence of the proposed treatment [21,31,32]. Similarly to other alkaline or mildly alkaline methods [15,16,33], the proposed treatment could remove from the fiber surface chemical components such as lignin, hemicellulose as well as wax and oils. Moreover, it could improve the fiber surface roughness as well as the number of cellulose chains exposed on the fiber surface, so that the number of bondings between the fiber surface and the polymeric matrix should increase [7,34].

As clearly shown in Figure 1 and Figure 2, it is worth noting that a worsening on the quasi-static properties of the treated composites is found when the concentration of the treating solution exceeds 10%. This means that, similar to the mercerization treatment [8,35], too high concentrations of the treating solution can negatively influence the fiber–matrix compatibility even for mildly alkaline treatment such as sodium citrate based one.

**Figure 5:**
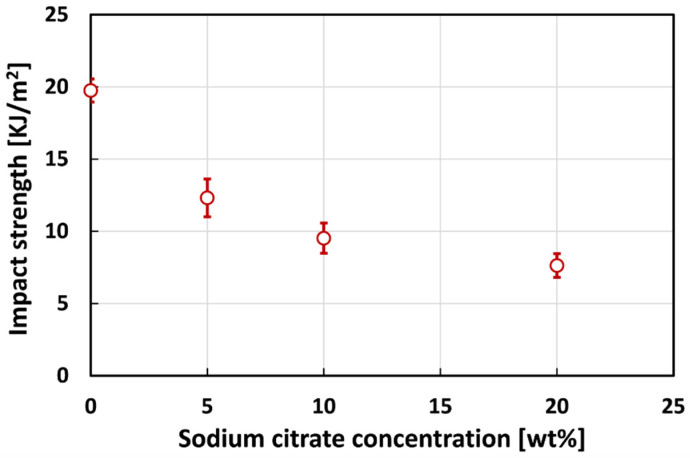
Impact strength of flax fiber reinforced composites at varying the sodium citrate concentration.

Figure 5 shows the effect of the concentration of the treating solution on the impact strength of the resulting composites. By observing Figure 5, it is possible to notice that the average values of the untreated samples (i.e., R composites) are higher than that of the treated composites, regardless the treatment conditions. In more details, the impact strength decreases by increasing the concentration of the treating solution: i.e., C5, C10 and C20 composites shows impact strength values of 37.7%, 51.8% and 61.4% lower than that of the reference, respectively (i.e., R composite).

**Figure 6:**
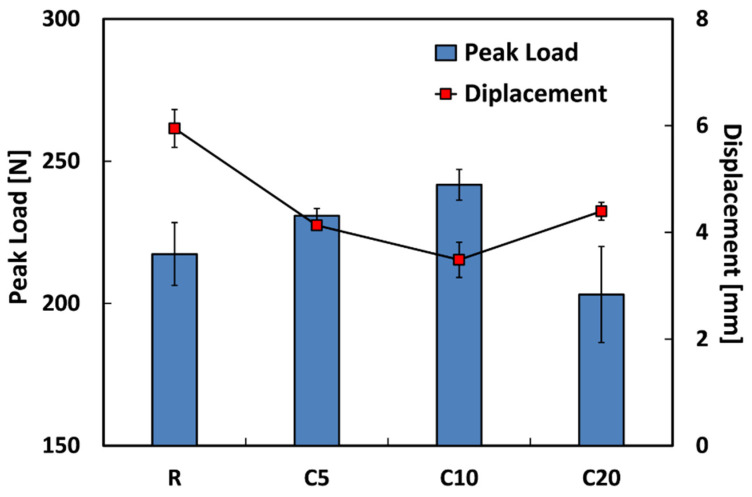
Peak load and maximum displacement values of flax fiber reinforced composites for each treatment condition.

With the aim of understanding this finding more deeply, it is of upmost importance to consider also the effect of the proposed treatment both on the peak load and on the maximum displacement. To this regard, the trends of these properties are depicted in Figure 6.

By observing this graph, it can be noticed that the impact strength increments shown by C5 and C10 composites in comparison to untreated one, are coupled both by improvements in their peak load and decrement in their maximum displacement (i.e., displacement at peak load). These results are in accordance with those obtained from quasi-static tensile and mechanical characterizations. In particular, it is confirmed the effectiveness of the proposed treatment on the compatibility between flax fibers and epoxy matrix, for concentrations of the treating solution up to 10%. Indeed, it is widely known that the improvement of the fiber–matrix adhesion due to natural fiber treatments also leads to a reduction of the impact strength of NFRC materials [19,36] by lowering the energy related to fiber pull-out phenomena. Moreover, the strongest interface between matrix and reinforcement phase surely allows the composites to improve their capacity of carrying load in addition to reduce their maximum displacement.

As shown in Figure 5, also 20% treated composites present, similarly to C5 and C10 composites, an average value of the impact strength noticeably lower than that of untreated composites (i.e., 7.63 KJ/m^2^ versus 19.75 KJ/m^2^). Differently from what happens for C5 and C10 composites, the reduction of the impact properties of C20 composites can be ascribed to the weakening of the fiber–matrix adhesion occurred when flax fibers are treated with 20% sodium citrate solution. This batch evidence average peak load value lower than R composites (i.e., 203.1 N versus 217.4 N), as shown in Figure 6. Furthermore, the maximum displacement shown by C20 composites is slightly lower than that of untreated composites but, at the same time, higher than that of C10 composites. All these findings confirm that the compatibility between natural fibers and polymeric matrix can be adversely influenced when the concentration of the treating solution is excessive (i.e., in this case higher than 10%).

**Figure 7:**
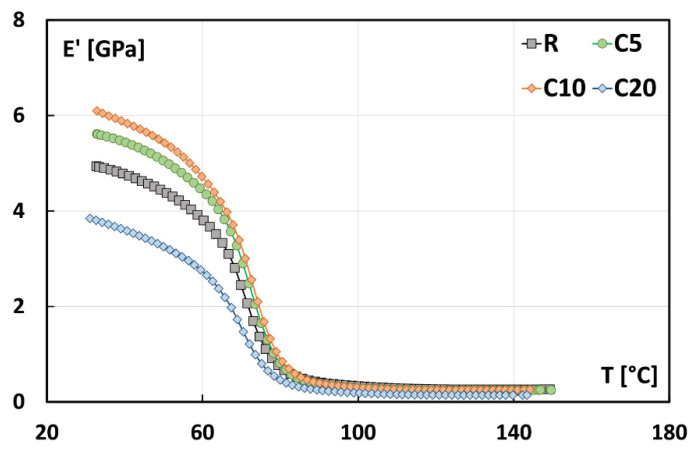
Typical storage modulus E’ trends of flax fiber reinforced composites.

Figure 7 shows the typical storage modulus (E’) versus temperature trends of untreated and treated flax fiber reinforced composites obtained through the DMTA characterization. By observing this graph, it can be noticed that each curve can be divided in two stages [37]. At temperatures lower than the glass transition temperature Tg of the polymeric matrix (i.e., named glassy region), all composites evidence a stiff behavior. In this region, fiber reinforced composites show high E’ values since both matrix and reinforcement are particularly immobilized. Vice versa, both components acquire mobility at temperature higher than Tg (i.e., rubbery region) and, as a consequence, a noticeable decrease of the storage modulus can be evidenced with increasing temperature.

As widely known the storage modulus E’ in the glassy region can be associated with the material stiffness: i.e., it measures the capability of composites to store the applied energy [38,39]. It is worth noting that the storage modulus values at 30 °C increases as function of the concentration of the treating solution up to 10% (Figure 7). On the contrary, C20 composites show E’ at 30 °C lower than that of R composites (i.e., 3.9 GPa versus 5 GPa). This trend is in accordance with the experimental results of quasi-static and impact characterizations, thus confirming the beneficial effect of the proposed treatment on the fiber–matrix compatibility when the concentration of the sodium citrate solution does not exceed 10% by weight.

**Figure 8:**
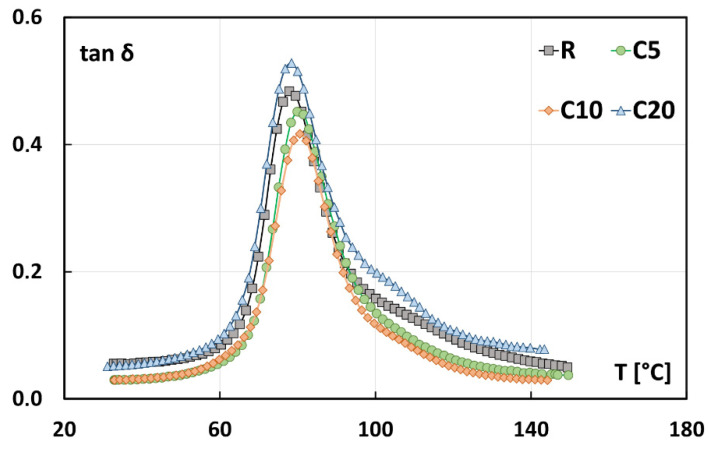
Typical tan δ trends of flax fiber reinforced composites.

Further information about the interfacial bonding between flax fibers and epoxy matrix can be acquired by analyzing the damping factor (i.e., tan δ) trends of composites shown in Figure 8. Damping factor is calculated as the ratio between loss modulus E’’ (representing the material tendency to dissipate the applied energy) and storage modulus E’. The shape of tan δ curves is strictly affected by the quality of the fiber–matrix interfacial bonding [37]. In particular, weak fiber–matrix interfaces lead to higher damping factor values. Conversely, a stronger adhesion limits the polymer chains mobility and, as a consequence, reduces the damping also shifting the Tg to higher values [38,40,41].

It can be noticed from Figure 8 that the tan δ peak values of C5 and C10 composites are lower than that of the untreated composites, due to the improved fiber–matrix compatibility. Conversely, an increase of this peak is noticeable for the 20% treated composites, indicating that C20 composite is characterized by a worse load carrying capacity in comparison to untreated one.

Furthermore, the Tg values of composites, calculated as the temperature at which the damping reaches its maximum value [42,43], are found in the narrow range between 77 °C and 81 °C, regardless of the treatment condition. In more detail, C5 and C10 composites show Tg average values equal to 80.4 °C and 80.3 °C, respectively, higher than that of untreated composites (i.e., 77.5 °C), thus evidencing once again the beneficial effect of the proposed treatment on the mechanical stability of the resulting composites. C20 composites show instead lower glass transition temperature (i.e., 78.3 °C) than other treated composites, which confirms that a weaker fiber–matrix interface is achieved if the concentration of the treating solution exceeds 10 wt.%.

### 3.2. Morphological Characterization

In order to correlate the mechanical response of composites with their morphology, the fractured surfaces of tensile specimens were observed with the aid of a scanning electron microscope (SEM).

**Figure 9:**
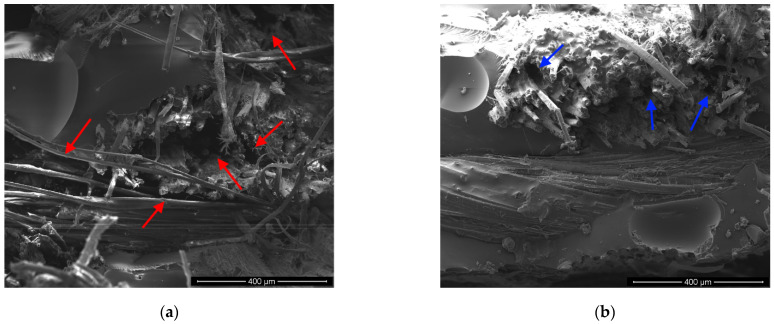

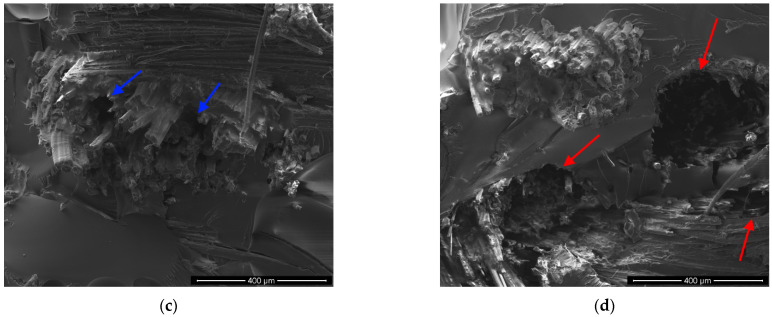
SEM micrographs at low magnification (250×) of tensile fractured surfaces: (**a**) R, (**b**) C5, (**c**) C10 and (**d**) C20 composites.

Figure 9 shows the morphologies at low magnification (250×) of the compared composites. In particular, several extended and large cracks (indicated by red arrows) can be visible at the fiber–matrix interfaces in the fractured surface of R sample (Figure 9a). These gaps are mainly due to the triggering and propagation of debonding and pull-out phenomena. Hence, this morphology clearly indicates a weak fiber–matrix adhesion [44,45], due to the low compatibility between the hydrophobic epoxy matrix and the hydrophilic untreated flax fibers. Furthermore, it fully justifies the poor mechanical properties shown by the epoxy composites reinforced with untreated flax fibers (i.e., R batch).

Conversely, the 5% and 10% treated samples evidence better morphologies than the untreated one, as evidenced in Figure 9b (i.e., C5) and Figure 9c (i.e., C10). Indeed, more compact structures without evident or large cracks can be observed in these images. Only few and small gaps (indicated by blue arrows) are visible in the fracture surface of C5 and C10 composites: i.e., fiber–matrix interfaces are not characterized by evident detachment zone. These findings evidence the effectiveness of the proposed treatment on the adhesion between flax fibers and epoxy matrix, when the concentration of the treating solution does not exceed 10%. Conversely, the morphology of the tensile fractured C20 samples (Figure 9d) clearly indicates that the proposed treatment is not effective when the concentration of the sodium citrate solution is too high. This figure shows the presence of large voids due to the absence of some fiber bundles as consequence of the occurrence of pull-out phenomena. As already stated in the previous sections, the poor fiber–matrix compatibility can be considered the main responsible for the scarce mechanical performances observed for this batch. These results clearly confirm that the concentration of the treating solution of 10% represents the optimum to improve the fiber–matrix adhesion and interface quality.

**Figure 10:**
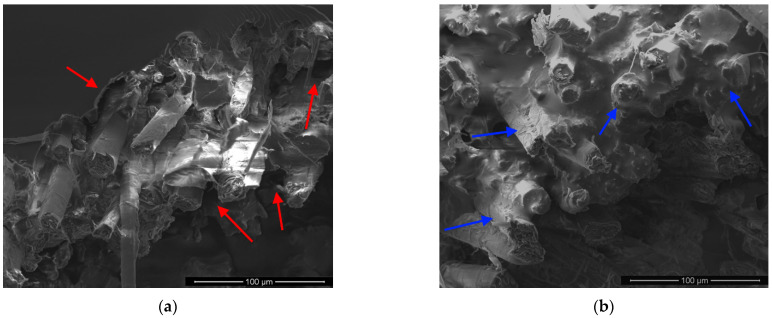

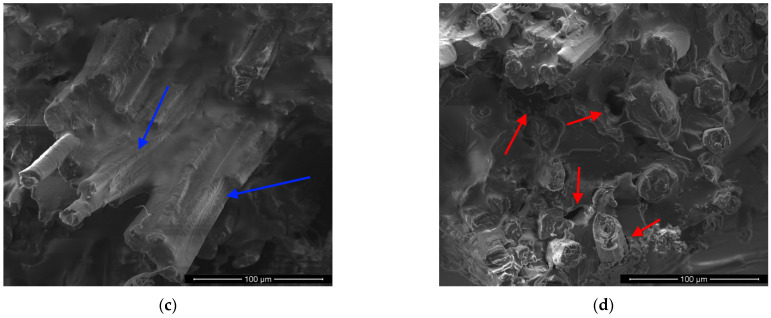
SEM micrographs at high magnification (1000×) of tensile fractured surfaces: (**a**) R, (**b**) C5, (**c**) C10 and (**d**) C20 composites.

In order to deeper analyze the effect of the proposed treatment on the fiber–matrix adhesion, the micrographs at high magnification (1000×) of the fractured surfaces of R, C5, C10 and C20 composites are shown in Figure 10.

By observing the morphologies of R and C20 composites (Figure 10a,d), it can be noticed that flax fibers are not well surrounded by the epoxy resin: i.e., some poor resin areas (indicated by red arrows) can be identified on the reinforcement surface, thus indicating an unsuitable fiber wettability. It is widely known that such kind of morphologies result in a high risk of debonding and pull-out events thus justifying the premature failures at low stress levels experienced by these composites [9,46].

On the contrary, a very high degree of matrix adhesion on fiber surface can be observed from the SEM images reported in Figure 9b,c, related to C5 and C10 composites. In particular, it is evident that the surface of flax fibers is covered by a continuous and densely packed layer of epoxy resin (blue arrows). This confirms that, if the concentration of the treating solution does not exceed 10%, the proposed treatment can be used in the production of high quality NFRC materials.

Overall, the morphological analysis evidences that the concentration of the treating solution equal to 10% is the optimum to improve the fibers-matrix adhesion and interface quality, with beneficial consequence on the mechanical response of the resulting composites.

As shown in Table 2, the main findings from the SEM analysis are fully confirmed by the void content’s assessment. It is commonly known that voids are the most common manufacturing-induced defects, which greatly influence both physical and thermomechanical properties of composite structures [47]. The quality level of composites can be assessed depending on its voids content [48]: i.e., a composite material can be defined excellent (voids content lower than 1%), good (voids content in the range between 1% and 5%) or poor (void content higher than 5%). The results shown in Table 2 show that the proposed treatment slightly influences the fiber volume fraction (i.e., in the range 36 ÷ 37%). More importantly, it is worth noting that the compared composites evidence a noticeable variation of their voids content due to the fiber treatment. In particular, the proposed treatment reduces the voids inside the composite structure, if the concentration of the treating solution does not exceed 10%. Conversely, C20 composites show the highest voids content (i.e., 7.2%) among the compared composites, thus confirming that too high concentration negatively affects the morphology of the produced composites.

**Figure 11:**
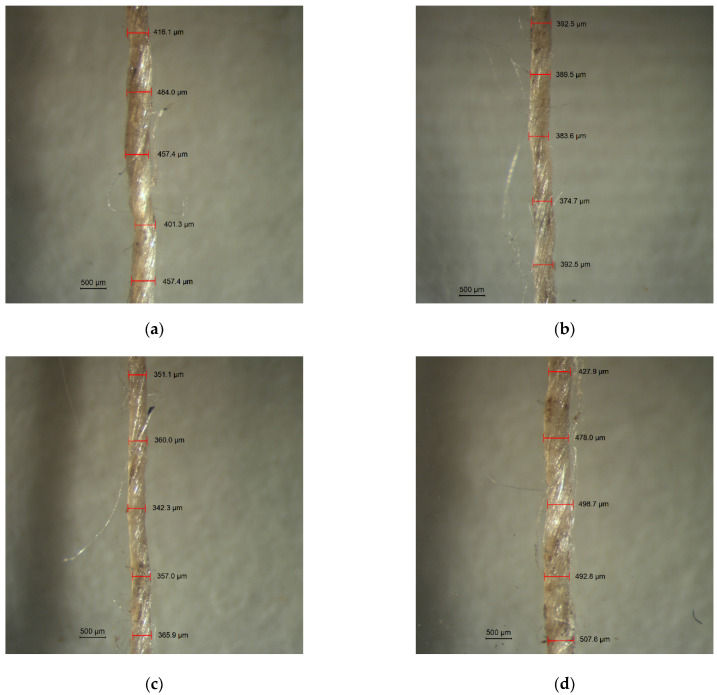
Optical micrographs of flax fibers for each treatment condition: (**a**) R, (**b**) C5, (**c**) C10 and (**d**) C20 fibers.

Moreover, it is important to note that the voids content of C5 and C10 composites is within the range 1 ÷ 5%, so that these materials can be both considered as good quality composite structures. On the other hand, R and C20 composites can be considered as poorly made materials since they exhibit too much voids (higher than 5%). These defects easy tend to form micro-cracks and, as a consequence, to reduce the mechanical properties of such composites. As previously stated, the experimental density values of flax fibers were measured by helium picnometry for each treatment condition. In detail, the average density value of untreated fibers is found to be 1.5020 g/cm^3^ whereas fibers soaked in 5%, 10% and 20% sodium citrate solutions show densities equal to 1.5139 g/cm^3^, 1.5187 g/cm^3^ and 1.5542 g/cm^3^, respectively. Hence, the fiber density increases with the concentration of the treating solution. This result can be explained by considering the modifications induced by the sodium citrate treatment on both fiber thickness and chemical structure of the fiber surface. In particular, it is worth noting that the fiber diameter decreases after the treatment due to fiber fibrillation, when the concentration of the treating solution increases up to 10% (Figure 11).

Conversely, the diameter of 20% treated fibers is found to be the highest, due to the occurrence of fiber swelling as well as salt crystals deposition over the fiber surface for this treatment condition. This means that the proposed treatment worsens the fiber structure when the concentration of the sodium citrate solution is too high.

**Figure 12:**
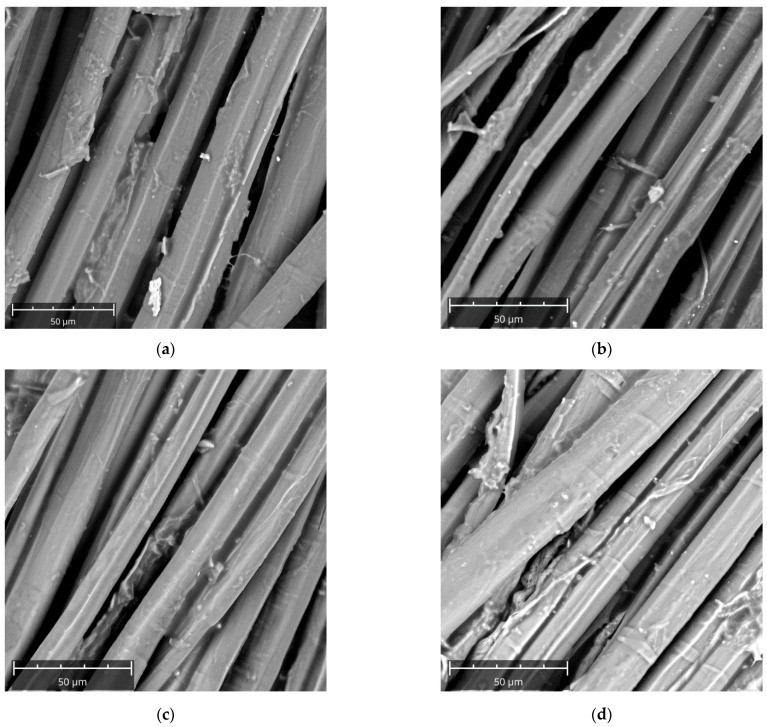
SEM micrographs of flax fibers for each treatment condition: (**a**) R, (**b**) C5, (**c**) C10 and (**d**) C20 fibers.

Similar findings can be acquired from SEM images of untreated and treated flax fibers (Figure 12). In particular, elementary fibers (i.e., microfibrils) bounded together by a lignin–hemicellulose matrix can be visible in Figure 12, regardless the treatment condition. For untreated fibers (Figure 12a), microfibrils show a rough surface, with presence of some impurities and absence of fiber fibrillation. On the other hand, C5 and C10 treated fibers (Figure 12b,c) show smoother surface, less impurities and reduced diameter, due to the removal of a certain portion of hemicellulose and lignin, as well as fiber fibrillation. Figure 12d confirms that, if the concentration of the treating solution exceeds 10 wt%, the microfibrils increase their diameter due to the occurrence of swelling phenomenon in addition to the deposition of some salt crystal over their surface.

**Figure 13:**
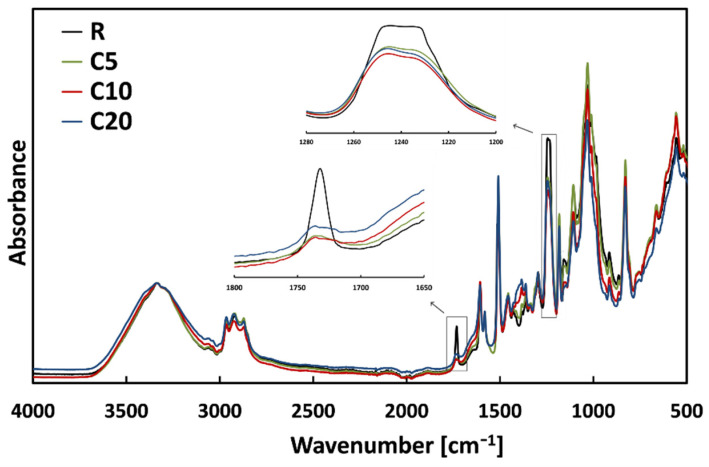
FTIR spectra of untreated and treated flax fibers.

On the other hand, the diameter reduction experienced by 5% and 10% treated fibers can be attributed to the modification induced by the proposed treatment on the chemical structure of the fiber surface, as shown by FTIR spectra of untreated and treated flax fibers (Figure 13). As suggested by literature, all the spectra were baseline corrected to the peak centered at 3337 cm^−1^, attributed to the O-H stretching vibration and hydrogen bond of the hydroxyl groups [16,49,50]. The spectra comparison clearly evidences that, similar to the traditional mercerization, the proposed treatment causes the removal of a certain portion of hemicellulose and lignin from the surface of fibers. Indeed, it is worth noting that the peak centered at 1732 cm^−1^, attributed to the carbonylic group C=O stretching vibration of linkage of carboxylic acid in lignin or ester group in hemicellulose, tends to disappear in the treated flax fibers [11,20,33,51]. Furthermore, a reduction of the peak centered at 1240 cm^−1^, due to the C-O stretching vibration of the acetyl group in lignin, can be noticed in the spectra of treated fibers [33,50,52]. No further noticeable changes can be appreciated in the spectra of treated fibers.

In addition to the diameter reduction, the partial removal of hemicellulose and lignin increases the number of possible reaction sites of cellulose available, thus favoring the adhesion with the polymeric matrix [53].

## 4. Conclusions

In the present paper, flax fibers were treated with solutions of sodium citrate (Na_3_C_6_H_5_O_7_) at different weight concentration (0–20%) to evaluate the effect of this innovative, cost-effective and eco-friendly treatment on the morphology and mechanical properties of epoxy based composites.

Fourier transform infrared analysis (FTIR) was carried out to evaluate the modifications on flax fibers surface induced by the proposed treatment. The morphology of the epoxy composites was analyzed with the aid of scanning electron microscope (SEM) and helium pycnometer whereas the composites mechanical response was evaluated by means of quasi-static tensile and flexural tests, Charpy impact tests and dynamical mechanical thermal tests.

The experimental results suggest that low concentrations are highly beneficial for improving the surface properties of flax fibers, thus enhancing their adhesion with the epoxy matrix. Indeed, composites reinforced with flax fibers treated in 10% sodium citrate solution showed the highest mechanical performances as well as the lowest porosity. On the other hand, higher concentrations led to fiber swelling, thus negatively affecting the composites morphology and, as a consequence, their mechanical properties.

In conclusion, it is possible to state that the proposed approach can be considered as an alternative to more expensive and harmful chemical treatments such as mercerization in the production of high quality NFRC materials.

## Figures and Tables

**Table 1:** Statistical results of the mechanical properties obtained from ANOVA.

		SQ	DF	MS	F	*p*-Value
**Tensile Strength**	Batch	2281.83	3	760.6099	257.9597	9.37727 × 10^−14^
	Error	47.17697	16	2.948561		
	Total	2329.007	19			
**Tensile Modulus**	Batch	13.87749	3	4.62582860	60.85088	5.71951 × 10^−9^
	Error	1.216305	16	0.07601908		
	Total	15.09379	19			
**Flexural Strength**	Batch	414.2694	3	138.0898	8.629053	1.22916 × 10^−3^
	Error	256.0463	16	16.00289		
	Total	670.3157	19			
**Flexural Modulus**	Batch	2.443777	3	0.814592295	11.70749	2.6105 × 10^−4^
	Error	1.11326	16	0.069578719		
	Total	3.557036	19			

**Table 2:** Main physical properties of composites for each treatment condition.

Sample	R	C5	C10	C20
Thickness [mm]	4.07 ± 0.02	4.44 ± 0.03	4.55 ± 0.07	4.71 ± 0.04
Theoretical density [g/cm^3^]	1.3183	1.3327	1.3334	1.3595
Experimental density [g/cm^3^]	1.2288	1.2941	1.3047	1.2610
Fiber content [%]	36.8 ± 0.2	36.5 ± 0.2	37.3 ± 0.5	36.2 ± 0.3
Voids content [%]	6.8 ± 0.1	2.9 ± 0.1	2.2 ± 0.1	7.2 ± 0.2

## Data Availability

The data has been obtained from the experimental results.
